# Supplementary material for: Neuronal changes and cognitive deficits in a multi-hit rat model following cumulative impact of early life stressors
Source: Biol Open. 2020 Sep 24;9(9):bio054130. doi: 10.1242/bio.054130 (PMC7522020; doi:10.1242/bio.054130)
Supplement: Supplementary information [file biolopen-9-054130-s1.pdf]

Sl. No.	Distance from the Soma (µm)	Interaction between HP control and treatment						Interaction between diet and treatment			
		LP+Poly I:C+LPS	LP+LPS	LP+Poly I:C	HP+Poly I:C+LPS	HP+LPS	HP+Poly I:C	HP vs. LP	HP+Poly I:C vs. LP+Poly I:C	HP+LPS vs. LP+LPS	HP+Poly I:C+LPS vs.LP+Poly I:C+LPS
1	20	$F_{(7,280)}$ =8.1, P≤0.001	NS	NS	NS	$F_{(3,210)}$ =6.07, P≤0.001	$F_{(3,210)}$ =8.102, P≤0.001	$F_{(1,210)}$ =3.5, P=0.013	$F_{(1,210)}$ =3.5, P=0.012	$F_{(1,210)}$ =3.56, P=0.012	$F_{(1,210)}$ =12.29, P≤0.001
2	40	$F_{(7,280)}$ =10.1, P≤0.001	$F_{(7,280)}$ =4.3, P=0.045	NS	$F_{(3,140)}$ =5.7, P≤0.001	$F_{(3,140)}$ =5.78, P≤0.001	$F_{(3,140)}$ =7.23, P≤0.001	NS	$F_{(1,210)}$ =4.6, P≤0.001	NS	NS
3	60	$F_{(7,280)}$ =8.1, P≤0.001	NS	$F_{(7,280)}$ =10.25, P≤0.001	$F_{(3,140)}$ =6.1, P≤0.001	-	$F_{(3,140)}$ =10.25, P≤0.001	NS	$F_{(1,210)}$ =4.78, P≤0.001	-	NS
5	80	$F_{(7,280)}$ =4.6, P=0.024	-	$F_{(7,280)}$ =11.539, P≤0.001	$F_{(3,140)}$ =11.5, P≤0.001	-	$F_{(3,140)}$ =9.23, P≤0.001	NS	$F_{(1,210)}$ =3.47, P=0.43	-	NS
6	100	-	-	$F_{(7,280)}$ =5.72, P≤0.001	$F_{(3,140)}$ =8.5, P≤0.001	-	$F_{(3,140)}$ =11.45, P≤0.001	NS	NS	-	-
7	120	-	-	NS	$F_{(3,140)}$ =17, P≤0.001	-	$F_{(3,140)}$ =8.52, P≤0.001	-	NS	-	-
8	140	-	-	NS	-	-	$F_{(3,140)}$ =10.64, P≤0.001	-	NS	-	-
9	160	-	-	-	-	-	-	-	-	-	-
10	180	-	-	-	-	-	-	-	-	-	-

NS=Non Significant
-= No values

Table S1
